# Internal Iliac Artery Aneurysm Ruptures with No Visualized Endoleak 2 Years after Endovascular Repair

**DOI:** 10.3400/avd.cr.21-00019

**Published:** 2022-03-25

**Authors:** Ayumi Harada, Koichi Morisaki, Shun Kurose, Shinichiro Yoshino, Sho Yamashita, Tadashi Furuyama, Masaki Mori

**Affiliations:** 1 Department of Surgery and Science, Graduate School of Medical Sciences, Kyushu University, Fukuoka, Fukuoka, Japan

**Keywords:** isolated internal iliac artery aneurysm, endovascular repair, endotension

## Abstract

We report a case of an 83-year-old man with a ruptured internal iliac artery (IIA) aneurysm after endovascular repair, which was treated via the ligation of IIA and tight suture of the aneurysm sac. Although there were no findings of obvious endoleak after endovascular treatment, the IIA aneurysm increased in size and eventually ruptured. We presumed that pressure to IIA aneurysm via the embolized IIA led to rupture. Aneurysm sac expansion may lead to a rupture despite no endoleak being detected; therefore, close follow-up or re-intervention must be considered. Tight embolization of IIA may prevent endotension in the same case.

## Introduction

Isolated iliac artery aneurysm constitutes <2% of all intra-abdominal aneurysms. Isolated internal iliac artery (IIA) aneurysm is even more unusual with an incidence of 0.4% of all intra-abdominal aneurysms.^1)^

Repair of iliac artery aneurysm is recommended when the iliac artery aneurysm diameter reaches 3.5 cm in the European Society for Vascular Surgery guideline.^2)^ Open surgery is the conventional treatment, but endovascular options are widespread recently because of a less invasive approach, usually by coils or plug embolization, stent graft placement, or a combination of both. Endovascular treatment is expected to decrease perioperative and postoperative morbidity and mortality rates.^3)^ However, endovascular repair often requires re-intervention compared with open repair.^4)^

As far as we know, there is no report regarding IIA aneurysm rupture due to endotension. We describe a case of IIA aneurysm rupture due to endotension 2 years after endovascular repair.

## Case Report

**Figure 1:**
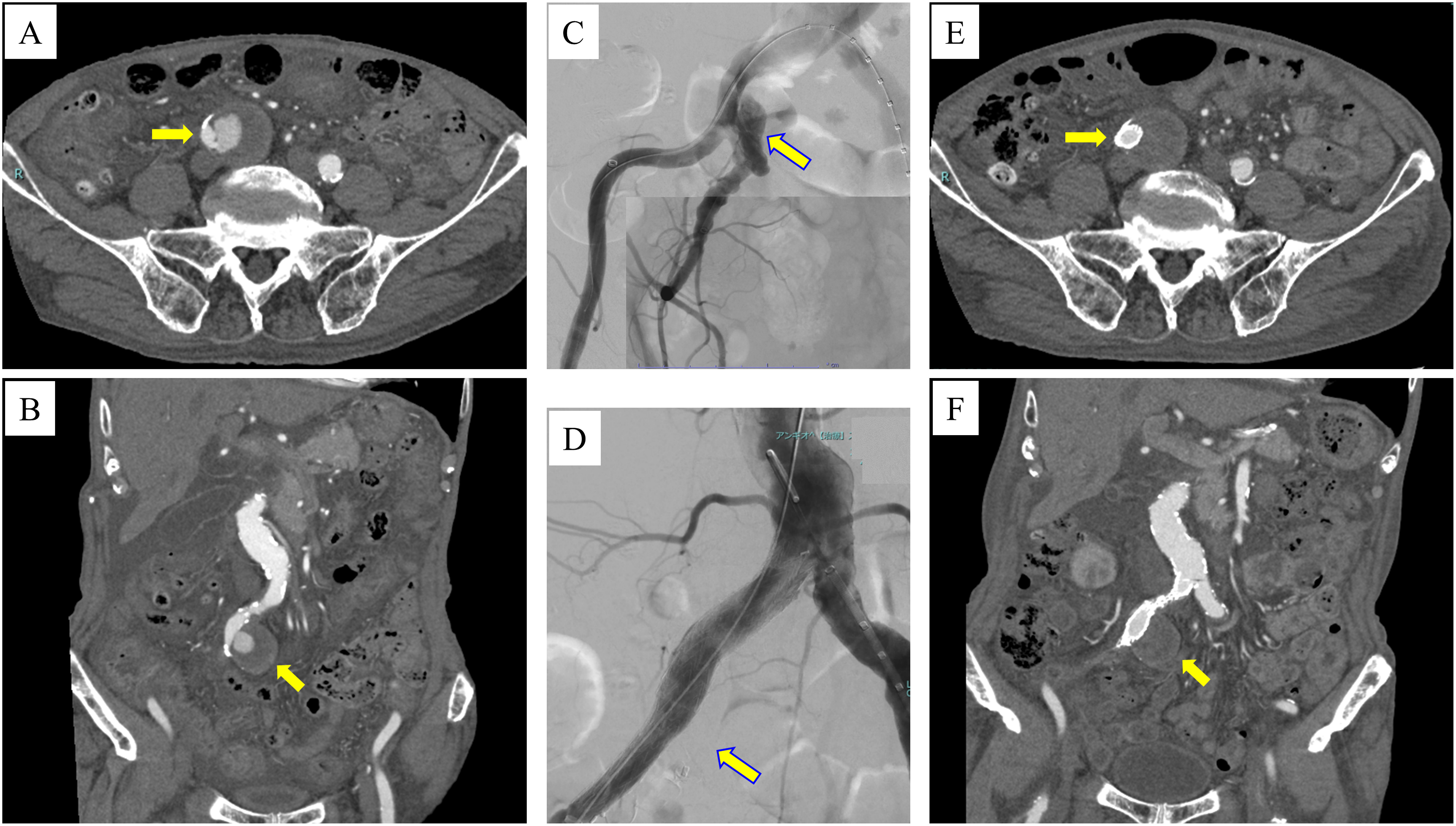
Fig. 1 (**A**), (**B**) Computed tomography (CT) showing a 36 mm right internal iliac artery (IIA) aneurysm. (**C**) Preoperative angiography showed IIA aneurysm. (**D**) Angiography at initial treatment after IIA embolization and stent graft placement showed no endoleak. (**E**), (**F**) The right IIA aneurysm was treated by IIA embolization and stent graft placement. CT 5 days after endovascular treatment showed no obvious endoleak.

An 83-year-old male presented with a right IIA aneurysm of 36 mm in diameter (**Figs. 1A** and **1B**). His comorbidities included hypertension, atrial fibrillation, and chronic heart disease, and he had taken warfarin. He underwent endovascular repair for the treatment of IIA aneurysm, including insertion of a stent graft from the common to the external iliac artery (PXC121000, W. L. Gore & Associates, Inc., Flagstaff, AZ, USA) following embolization of the right IIA using an AMPLATZER Vascular Plug II (9-AVP2-012, St. Jude Medical Inc., St. Paul, MN, USA) (**Figs. 1C** and **1D**). No endoleak was seen on computed tomography (CT) 5 days after the endovascular repair (**Figs. 1E** and **1F**).

**Figure 2:**
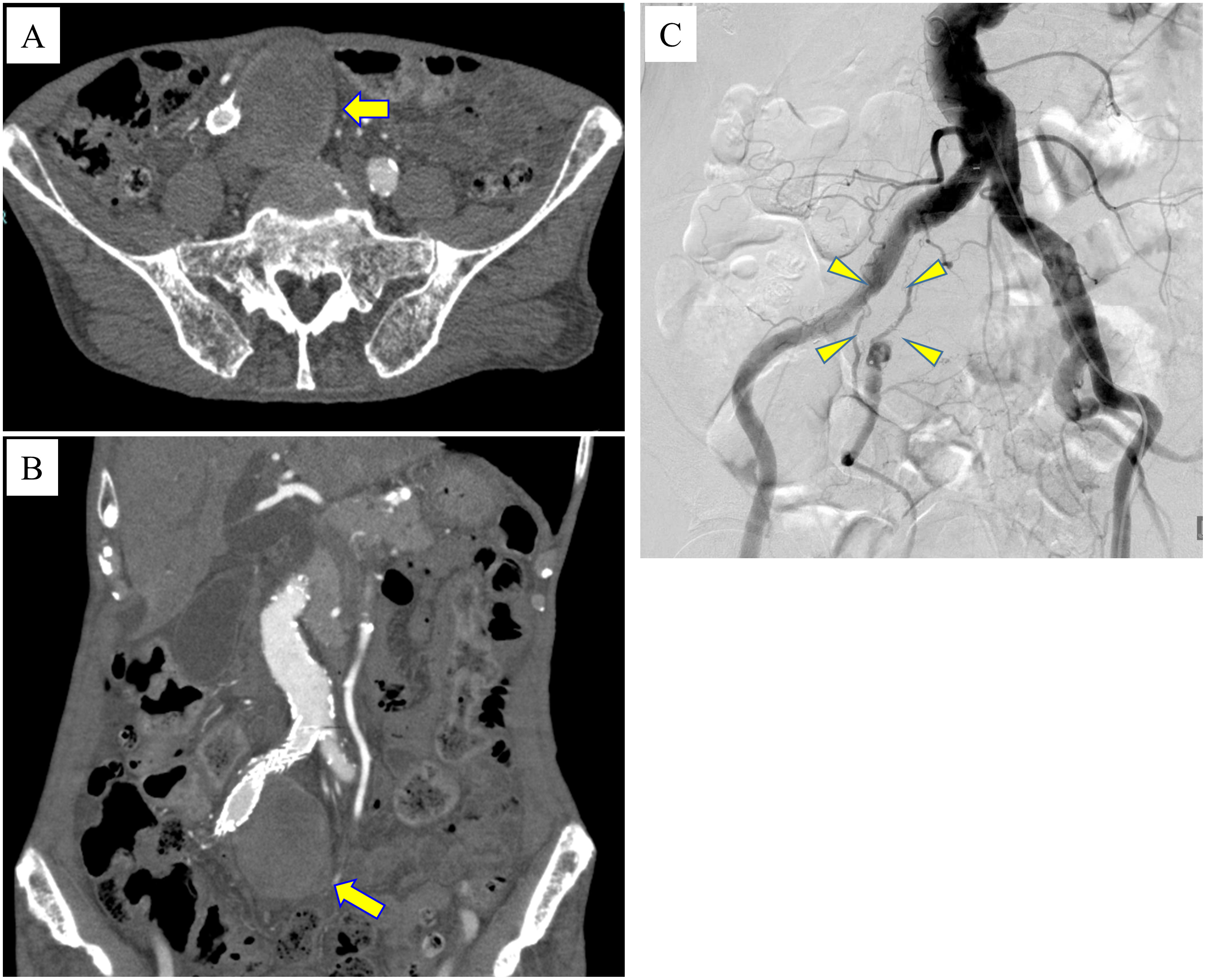
Fig. 2 (**A**), (**B**) Computed tomography 2 years after endovascular treatment showing a 50 mm right internal iliac artery aneurysm with no obvious endoleak. (**C**) Angiography showed no findings of endoleak 2 years after endovascular repair.

Two years after the first treatment, CT revealed that the aneurysm gradually expanded to a diameter of 50 mm without evidence of endoleak (**Figs. 2A** and **2B**). Angiography showed no endoleak (**Fig. 2C**). Considering advanced age and his comorbidities, further intervention was not offered at that time due to no findings of endoleak.

**Figure 3:**
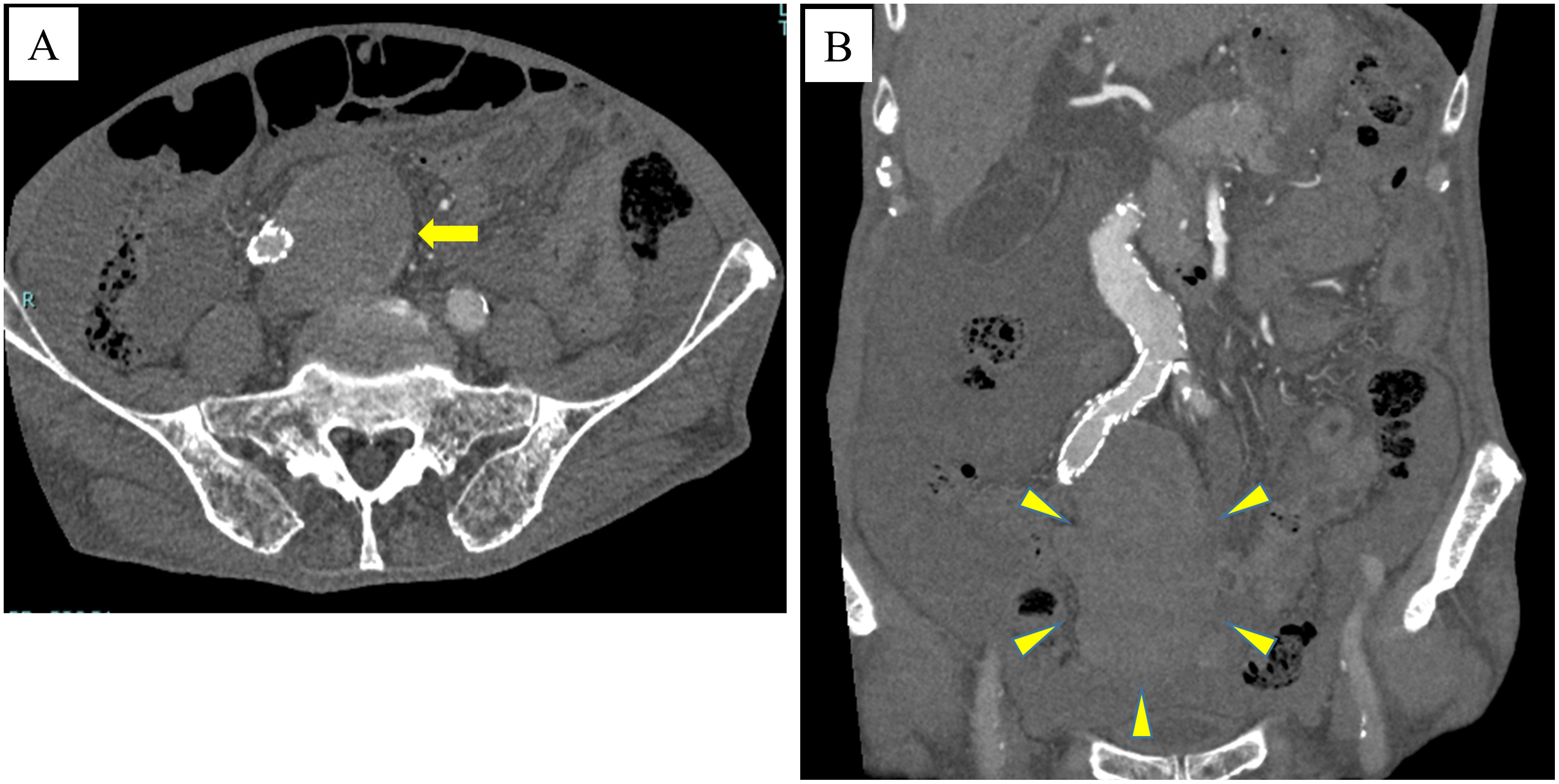
Fig. 3 (**A**), (**B**) Computed tomography showing a ruptured internal iliac artery aneurysm (arrow) and a large hematoma around the aneurysm (arrowheads).

One month after, the patient took a blood test regularly because of the administration of the oral intake of warfarin. Although the patient had no symptoms such as abdominal or back pain, anemia was detected via the blood test. His hemoglobin level dropped from 10.3 to 7.5 g/dL, and prothrombin time–international normalized ratio (PT-INR) was markedly elevated to 3.51. Serum creatinine level was 1.10 mg/dL, and no data were suggesting disseminated intravascular coagulation. Upper gastrointestinal endoscopy showed no findings of gastrointestinal bleeding. Thus, the patient underwent a CT examination to explore the cause of anemia. Although CT showed a hematoma around the right IIA aneurysm, there are no findings of endoleak (**Fig. 3**). His vital signs were stable (blood pressure, 102/64 mmHg; heart rate, 68 beats per minute).

Elective open surgery was performed via retroperitoneal approach under general anesthesia. A ruptured hole was detected at the anterior of the right IIA aneurysm. Common and external iliac arteries were not clamped. The aneurysm sac was incised, and the intraluminal thrombus was removed. There were no findings of obvious endoleak. A fresh hematoma around the aneurysm was removed as much as possible. The AMPLATZER Vascular Plug II, which had been deployed to right IIA in previous endovascular treatment, was removed, and backflow from IIA was detected. Distal IIA was sutured and aneurysm sac was sutured tightly. His anemia was improved, and CT showed no expansion of hematoma after the open surgery.

## Discussion

As far as we know, there is no previous report regarding the rupture of IIA aneurysm after endovascular repair due to endotension. Bade et al. reported IIA aneurysm rupture due to endotension.^5)^ However, open surgical repair revealed the cause of the rupture was a type 2 endoleak from a small branch vessel in that case. Ruurda et al. reported two cases of IIA aneurysms expansion caused by endotension after surgical exclusion of the inflow. Although CT or angiography showed the absences of endoleak, back bleeding from side branches were found at open surgery.^6)^ Back pressure of collateral circulation may lead to sac growth despite no endoleak being detected. In the present case, endoleak was not detected via CT or angiography. Habets et al. reported the effectiveness of magnetic resonance imaging (MRI) for the detection of endoleaks in a systematic review.^7)^ Although MRI was not performed in the present case, MRI probably could not detect endoleak because no endoleak was detected in open surgery. Moreover, endotension from embolized IIA or excessive anticoagulation may lead to rupture in the present case. However, there is no obvious evidence to support these hypotheses.

Proximal and distal neck diameter was 13–14 and 9 mm, respectively. Proximal and distal landing zones were at least 3 cm at initial endovascular repair. Thus, we think proximal and distal landing zones were adequate. The diameter of distal IIA was 8–9 mm, therefore IIA was embolized with AMPLATZER Vascular Plug II 12 mm at initial endovascular repair. Although any endoleak including type 2 endoleak from embolized IIA or branch vessels was not detected when ruptured aneurysm sac was incised, the vascular plug was removed and distal IIA was sutured at the open surgery, considering the possibility of endotension from embolized IIA. These treatments may not resolve the sac expansion and removal of stent graft with replacement using prosthetic graft is another treatment option. In the present case, less invasive treatment was selected considering advanced age and the patient’s comorbidities.

Sahgal et al. reported the case of common–internal iliac artery aneurysm rupture after endovascular repair despite no findings of endoleak and shrinkage of the aneurysm sac were detected.^8)^ In that case, IIA was embolized at part of a thrombus-lined, which caused aneurysm rupture by intra-aneurysmal pressure through the clot from IIA branches.

Excessive anticoagulation may affect the rupture of IIA aneurysm, increasing the intraluminal pressure from embolized IIA.^9)^ Iyer et al. reported aneurysm sac enlargement due to endotension associated with excessive warfarin anticoagulation. Aneurysm sac regressed after reversal of excessive anticoagulation. In the present case, though distal IIA was embolized at the normal part, prolonged PT-INR via warfarin therapy may influence the intraluminal pressure through the embolized IIA, which may lead to aneurysm rupture.

Some literatures showed that anticoagulant administration can be associated with an increased risk for persistent type 2 endoleak after endovascular repair. However, there is no consensus on the relationship between anticoagulant therapy and the increased frequency of type 2 endoleak currently.^10)^ Furthermore, the relationship between anticoagulant administration and treatment outcome of embolization for the prevention of type 2 endoleak remains unclear.

Dorenberg et al. reported recanalization of the AMPLATZER Vascular Plug (AVP), which lead to recurrent IIA aneurysm rupture. He proposed a combination of the AVP with coils in the same case.^11)^ In the present case, pressure through the embolized IIA may lead to aneurysm rupture despite no findings of endoleak. Thus, tight embolization such as AVP with coils may prevent endotension. Although right IIA was initially embolized to preserve the collateral circulation, embolization of gluteal arteries at the first endovascular treatment may also prevent endotension.

## Conclusion

We report a rare case of IIA aneurysm rupture due to endotension after endovascular repair. Aneurysm sac expansion may lead to rupture despite no findings of endoleak; therefore, close follow-up or re-intervention should be considered. Tight embolization such as AVP with coils or embolization of each branch of IIA may prevent endotension in the same case.
